# Effects of Neural Correlates of Food-Specific Intentional Inhibition in Predicting Body Fat Loss for Overweight and Normal-Weight Young Adults: The Mediation of Restrained Eating

**DOI:** 10.3390/nu18111670

**Published:** 2026-05-23

**Authors:** Xinyuan Liu, Mingzhu Li, Shiqing Song, Yicen Cui, Hong Chen

**Affiliations:** 1 Faculty of Psychology, Hebei Normal University, Shijiazhuang 050024, China; liuxiny@hebtu.edu.cn (X.L.);; 2 Faculty of Psychology, Shaanxi Normal University, Xi’an 710062, China; 3 Faculty of Psychology, Southwest University, Chongqing 400715, China

**Keywords:** overweight, food-specific intentional inhibition, restrained eating, fMRI, relative fat mass

## Abstract

**Background/Objectives**: Intentional inhibition reflects voluntary control abilities and is assumed to be an indicator of overweight. The medial frontal cortex is an important brain region associated with intentional inhibition. Nevertheless, it is uncertain whether being overweight is connected to impaired food-related intentional inhibition (FII), and if so, what its underlying neural correlates are. The present study therefore aims to provide increased support for overweight due to impairment of FII. **Methods**: Firstly, 55 overweight and 45 normal-weight college students (Sample 1) were instructed to perform a go/no-go/choose task, which included a resting-state fMRI. Neural correlates of FII were examined using regional homogeneity (ReHo) analyses. Subsequently, an additional 180 undergraduates (87 overweight and 93 normal-weight; Sample 2) were examined to ascertain the differences in resting-state functional connectivity (rsFC) between overweight and normal-weight participants. The study also investigated whether restrained eating mediated the effect of rsFCs on one-year body index changes. **Results**: FII demonstrated a positive correlation with the cerebellum, inferior temporal gyrus, orbitofrontal cortex, inferior frontal gyrus, and cingulate gyrus. Additionally, in comparison with participants with normal weight, overweight participants demonstrated diminished rsFC between the FII-related areas and the postcentral gyrus, while heightened rsFC strengths were found between these areas and the middle temporal gyrus and precuneus. Furthermore, mediation analyses demonstrated that cingulate–precuneus connectivity is linked to fat mass index change a year later through restrained eating. **Conclusions**: FII was associated with connectivity between brain regions involved in inhibitory control and maladaptive eating. Furthermore, we investigated how these connectivity patterns could potentially affect future body fat loss through restrained eating.

## 1. Introduction

The issue of overweight/obesity has emerged as a significant public health problem [1]. The World Health Organization (WHO) estimates that the percentage of overweight/obese adults worldwide is as high as 40%, and this value is expected to reach 58% by 2030. There is robust evidence indicating that overweight/obesity contributes to the development of many chronic diseases, such as hypertension and cardiovascular disease [2]. In addition, there is evidence that excess weight and overconsumption of high-calorie foods have adverse effects on brain development and executive function [3]. In the context of tasks related to food, overweight/obese individuals have been observed to demonstrate more poor inhibitory control. However, they have also been found to have a heightened sensitivity to food-related stimuli [4]. However, in previous theoretical and empirical studies of inhibitory control in overweight/obese individuals, researchers have largely focused on reactive inhibition (RI). This approach has been unable to reveal the unique characteristics of the processes involved in intentional inhibition [5].

Intentional inhibition is the capacity to voluntarily suspend or inhibit an action [6]. It focuses on the voluntary and internal decision to withdraw an action, in which respect intentional inhibition clearly differs from reactive inhibition. Based on the theoretical intentional inhibition model, intentional inhibition is a process that connects a distal goal with a proximal goal [7]. In combination with the model of conflicting goals in eating behavior [8], the proximate goal here can be understood as hedonic eating, whilst the distal goal is weight control. This perspective suggests that intentional inhibition plays a significant role in inhibiting maladaptive behaviors and selecting healthy behaviors. Consistent with this model, converging evidence has suggested associations between a lack of intentional inhibition and unhealthy behaviors, such as cigarette smoking [9], alcohol use [10], and food cravings [11,12]. In the case of eating behaviors, there are usually no clear external cues in daily life to prompt individuals to withdraw these behaviors. Therefore, investigating food-specific intentional inhibition (FII) in overweight individuals will help them achieve dietary control and weight management in real-life settings.

Neuroimaging research has consistently found that intentional inhibition is associated with the inferior frontal gyrus (IFG) and medial frontal cortex (MFC) [6,13]. The IFG plays a role in both intentional inhibition and reactive inhibition and is a key physiological basis for the effective execution of behavioral control [14]. Moreover, the IFG may be related to food-related inhibition. For example, in one study, in contrast to restrained eaters who received a sham intervention, those who received transcranial direct current stimulation (tDCS) exhibited elevated levels of inhibition when exposed to food cues during a stop-signal task [15]. The existing literature has also proposed that the IFG is positively linked to body mass index (BMI) [16] and negatively associated with predicting future weight gain [17].

In addition to the IFG, which overlaps with reactive inhibition, intentional inhibition has been demonstrated to also cause hyperactivity in the MFC [6,13]. To our knowledge, only one previous study has examined the resting-state functional connectivity (rs-FC) of the MFC in overweight individuals [18]. The study measured blood-oxygen-level-dependent (BOLD) signals in rs-fMRI, which are thought to reflect neural oscillations and behavioral variability [18]. The results showed that, compared with the normal-weight group, the overweight group exhibited enhanced functional connectivity between the MFC and areas associated with maladaptive eating, such as the cerebellum, middle temporal gyrus (MTG), and postcentral gyrus (PCG). Moreover, a previous study found FII to be significantly positively associated with these rsFCs [18]. These findings suggest that areas associated with maladaptive eating are also involved in FII. Maladaptive eating behavior is defined as eating unhealthy food and not eating healthy food, including restrained eating and disinhibited eating [19,20,21,22]. Further, a recent meta-analysis found a positive correlation between BMI and the activity in the cerebellum, PCG, MTG [23], and cingulate gyrus [24]. In addition, it was found that MFC–cerebellum connectivity was a positive predictor of weight gain over one year in overweight individuals but not in normal-weight participants. Therefore, altered activation and connectivity of brain regions correlated with inhibitory control and maladaptive eating behavior may serve as a mechanism underlying the challenges encountered by overweight individuals who actively inhibit food-specific stimuli in their daily lives.

However, the neural basis of FII in overweight individuals is not clear. Based on the above evidence, this study posits that FII is related to the amplitude of ReHo in areas correlated with inhibition and maladaptive eating behavior. Noninvasive rs-fMRI is a powerful tool for the measurement of resting brain activity [25] and the investigation of the neural correlates of intentional inhibition [18]. Regional homogeneity (ReHo) analysis is a robust index [26] that effectively assesses resting-state brain activity [27]. It is hypothesized that brain activity occurs more commonly in clusters than in single voxels. To this end, ReHo is evaluated via Kendall’s coefficient of concordance (KCC) [28], which assesses the similarities between a given voxel’s time series and those of its nearest neighbors. Accordingly, ReHo has been demonstrated to reflect the local coherence of spontaneous neuronal activity [29] and to facilitate the unravelling of functional connectivity between brain regions [30]. Although rs-fMRI studies have examined FC for between-group differences in overweight and normal-weight individuals using regions related to intentional inhibition as seeds [18], there have been no extensions to date exploring the neural correlates of FII.

In addition, recent studies have highlighted the significance of restrained eating (RE) and the presence of inhibition deficits as a contributing factor to the perpetuation of obesity [31]. RE refers to a tendency for individuals to feel anxious about their food intake and to try to restrict it [32]. Some studies have found that overweight/obese participants only made more errors in the food-specific go/no-go task compared to lean participants when they had low dietary restraint [33]. In contrast, other research has illustrated that obese participants made more commission errors in response to food-related cues when they were restrained eaters [32]. To date, it is unclear how RE mediates the relationship between inhibitory control and overweight. The present study therefore aimed to explore the neural activity and connectivity underlying FII and how these neural patterns affect weight gain mediated by RE.

It is hypothesized that activity in the areas associated with inhibitory control (e.g., IFG) and maladaptive eating (e.g., cerebellum) might play a key role in FII (hypothesis 1). Furthermore, compared to normal-weight participants, overweight individuals are hypothesized to show reduced rsFCs between areas correlated with FII and maladaptive eating (hypothesis 2). In addition, RE might act as a critical mediator in the relationship between group-differential connectivity and change in body indexes (BMI, relative fat mass [RFM], waist-to-height ratio [WHtR]) one year later (i.e., rsFC → RE → body indexes) (hypothesis 3). Finally, this study comprehensively integrates the above findings to propose a food-specific intentional inhibition model for overweight.

## 2. Method

### 2.1. Participants

**Figure 1:**
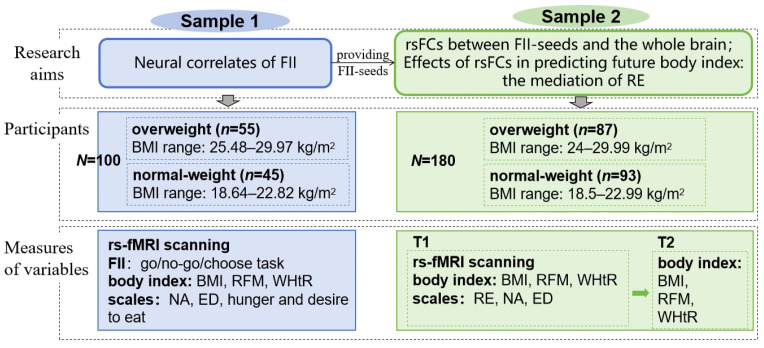
The flowchart of the study design. Note: FII, food-specific intentional inhibition; *N*, number; BMI, body mass index; RFM, relative fat mass; WHtR, waist/height ratio; NA, negative affect; RE, restrained eating; ED, eating disorder.

Two samples were included in this study. An analysis was conducted to identify the neural correlates of FII in Sample 1. In Sample 2, we tested connectivity between FII-seeds and the whole brain and examined the effects of these rsFCs in predicting future body indexes. See Figure 1 for the study design overview.

Sample 1 included 100 participants. The sample size was ascertained through a priori power calculations in G*Power 3.1. The estimated effect size (Cohen’s f) was 0.25, and a power was set at 0.90. It was determined that a minimum of 98 participants would be required to detect the effects of interest (alpha = 0.05). Sample 1 was recruited through campus advertisements and consisted of 55 overweight participants (27 males, mean BMI = 27.24 ± 1.45 kg/m^2^) and 45 normal-weight participants (24 males, mean BMI = 20.34 ± 1.16 kg/m^2^) aged 18–23 years. The data collected from them included body indexes (BMI, RFM, WHtR), NA, ED, hunger and desire to eat, resting-state fMRI scanning, and FII performance test outside the scanner.

Sample 2 included 180 participants (77 males; aged 18–22 years). Sample 2 was derived from the Behavioral Brain Research Project. In the first wave (T1, September 2019–January 2020), data were collected from 1020 college students regarding body indexes (BMI, RFM, WHtR), NA, RE, ED, and resting-state fMRI scanning. One year later, we tracked 567 original participants and collected their body indexes (T2, September 2020–January 2021). A total of 87 overweight participants (BMI > 24 kg/m^2^) completed all of the above data collection. Subsequently, 93 normal-weight participants (BMI: 18.5 < kg/m^2^ < 23) were matched with the overweight group on the basis of age and gender at the group level. A post hoc power analysis of ANCOVA, in which 180 participants provided 0.99 power by setting the alpha level at 0.05, determined a large-size effect (f = 0.30). Table 1 shows the demographic characteristics of the two groups in each of the two samples.

Participants were all right-handed college students from a university in Chongqing, China. None of the participants in this study had any eating disorders. Moreover, they reported no history of psychiatric or neurological illnesses, no previous long-term medication use within the past six months, and were in good physical health. Participants were instructed to abstain from eating, drinking alcohol, or consuming coffee for a period of 8 h prior to the measurements. Informed consent forms were signed by all participants prior to the commencement of the study. The present study received ethical approval from the Ethics Committee of the university.

### 2.2. Measures

#### 2.2.1. Body Indexes

The standard formula was utilized to calculate BMI: weight (kg)/height^2^ (m^2^). The formula for calculating RFM was: 64 − (20 × height/waist circumference) + (12 × gender). In this calculation, the gender value is 0 for men and 12 for women. The WHtR was calculated using the following formula: waist circumference (cm)/height (cm). The measurement of these body indexes was all undertaken with the utilization of the SECA medical human body component analyzer [11,34].

#### 2.2.2. Hunger and Desire to Eat

Participants rated their feelings of hunger and desire to eat using a 100 mm visual analog scale. The scale ranged from “not at all” to “very much.”

#### 2.2.3. Negative Affect

Positive and Negative Affect Schedule (PANAS) [35] is a questionnaire used to assesses current mood state, including negative and positive affects. PANAS comprises 20 items, with each rated on a 5-point scale ranging from 1 (not at all) to 5 (very much). Participants were asked to indicate the extent to which they currently felt each of the 10 adjectives measuring positive affect (α = 0.87) and 10 adjectives measuring negative affect (α = 0.89). In the present study, the negative effect score for this scale was calculated by summing the scores for the ten adjectives measuring negative effects.

#### 2.2.4. Restrained Eating

A Chinese version of the Restrained Scale [36] was used to evaluate RE. The revised scale contains 10 items. Participants were requested to evaluate their dietary habits in the present moment, using a 5-point scale from 0 (never) to 4 (always). Higher scores on this scale indicated a greater tendency toward RE. The Restrained Scale has shown good internal consistency and acceptable temporal reliability in Chinese samples [36]. In this study, Cronbach’s α = 0.76.

#### 2.2.5. Go/No-Go/Choose Task

The food-specific go/no-go/choose task was developed for the purpose of measuring FII in our previous studies [11]. The stimuli comprised 10 images of high-calorie food and 10 images of low-calorie food. The target stimuli comprised 3 types, each with an equal size perimeter of 6 cm. The first type was the go target, which was a square (50% of all trials). The second type was the no-go target, which was a circle (16% of all trials). The third type was the free-choice target, which was a diamond (34% of all trials). Each trial commenced with a “+” for 500 ms, followed by a target stimulus for 120 ms. Participants were asked to initiate a key-press action at the point at which “+” appeared and to respond to the shape of the stimuli. In go/no-go trials, participants were required to perform or withhold the key-press action. In free-choice trials, participants were asked to make a voluntary decision to either perform or inhibit the action. The experiment comprised one practice block of 18 trials and six test blocks of 720 trials, which were presented at random. Participants rested for 1–5 min between blocks.

According to previous research, in the free-choice task, there is no external cuing requiring participants to respond or inhibit a key-press action. Therefore, the proportion of responses in the free-choice task was used as the key indicator of FII [11,12]. In this study, the proportion of responses in the free-choice task was used as the indicator of FII, with higher FII values representing poorer intentional inhibition performance.

### 2.3. Imaging Data Acquisition and Preprocessing

#### 2.3.1. Imaging Acquisition

Each subject underwent an 8 min rs-fMRI scan on a 3 T Trio MRI scanner (Siemens Medical Systems, Erlangen, Germany). For each subject, 240 contiguous volumes were acquired using a gradient echo-planar imaging sequence. Scanning parameters were as follows: repetition time (TR) = 2000 ms; echo time (TE) = 30 ms; slices = 62; slice thickness = 2 mm; field of view read = 224 × 224 mm^2^; flip angle = 90°; resolution matrix = 112 × 112; voxel size = 2 × 2 × 2 mm^3^; phase encoding direction = PC ” AC.

During scanning, all participants were required to remain motionless, adopt a relaxed posture with their eyes closed, and avoid thinking consciously about anything. Foam pads and earplugs were used to minimize head motion and scanning noise. None of participants of this study exhibited head motion between volumes in any direction >2.0 mm or rotation in any axis >2.0 during scanning, or mean framewise displacement (FD) >0.50.

#### 2.3.2. Image Data Preprocessing

All preprocessing steps for the imaging data were performed with the CONN functional connectivity toolbox (version 20.b; https://www.nitrc.org/projects/conn [accessed on 22 January 2022]) in conjunction with SPM12 (http://www.fil.ion.ucl.ac.uk/spm [accessed on 22 January 2022]). The preprocessing procedure was as such: Initially, the first ten images were eliminated to enable participant familiarization and fMRI signal stabilization. The images that remained were corrected for temporal shifts between slices and realigned to the middle volume. Second, the images were spatially normalized to Montreal Neurological Institute (MNI) space with a resolution voxel size of 2 × 2 × 2 mm^3^. Then, they were spatially smoothed using a 4 mm full width at half maximum (FWHM) of the Gaussian kernel. This was followed by the removal of linear trends. Nuisance signals (comprising white matter, cerebrospinal fluid, and head-motion parameters) and their derivatives were regressed using a Friston 24-parameter model in order to control for potential physiological effects. Lastly, the implementation of data scrubbing addressed concerns regarding head motion. Problematic timepoints were considered as regression factors, defined as volumes with framewise displacement (FD) power >0.5 mm, along with the two subsequent volumes and one preceding volume, to minimize the spillover effect of head motion.

### 2.4. Data Analysis

#### 2.4.1. ReHo–Behavioral Correlation Analysis

In Sample 1, the DPABI software (version 4.3; http://rfmri.org/dpabi [accessed on 2 June 2022]) was used to conduct a whole-brain correlation analysis of FII and normalized ReHo values (i.e., z-ReHo) [26], while controlling for age, gender, and FD. The threshold for significant regions was set at cluster *p* < 0.05, voxel *p* < 0.005, dimensions 61 × 73 × 61, and two-tailed (GRF).

#### 2.4.2. ROI-Wise rsFC Analysis

Based on the results from the ReHo–behavioral correlation analysis, in Sample 2 (*N* = 180), 5 regions of interest (ROI) were generated as spherical seeds with a radius of 5 mm, based on the brain regions observed in the ReHo–behavioral correlation analysis [37,38]. The center of mass MNI coordinates were as follows: x = −48, y = 48, z = 6 (IFG); x = 69, y = −27, z = −21 (inferior temporal gyrus, ITG); x = 33, y = 42, z = −18 (orbitofrontal cortex, OFC); x = 18, y = 0, z = 30 (cingulate gyrus); and x = −42, y = −72, z = −33 (cerebellum). Time series data were extracted for each participant from all of the seeds.

For each ROI, whole-brain rsFC analysis was performed in order to identify disparities in brain connectivity between overweight and normal-weight participants. Connectivity analysis was carried out over the complete duration of an 8 min block [39]. The controlled variables included age, gender, and head motion. The resulting connectivity maps were subsequently subjected to Fisher-z transformation. Analyses were performed using SPM 12 and DPABI 4.3. The threshold for significant regions was set at *p* < 0.05 (cluster), *p* < 0.001 (voxel), and two-tailed (GRF).

#### 2.4.3. Prediction and Mediation Analyses

In this study, regression analyses were performed to explore the relationship between connectivity and future body indexes, including body indexes at T2 (BMI, RFM, WHtR) and the difference calculated as T2 minus T1 (ΔBMI, ΔRFM, ΔWHtR). Two-step regressions were conducted for each rsFC, with gender and age entered in the first step and all rsFCs entered in the second step. It was not possible to incorporate all rsFCs within a single model, as this introduces many unobserved possibilities into the model, thereby reducing the level of variance needed to facilitate the observation of model effects [40].

To ascertain whether RE provides a reliable explanation for the covariation between rsFCs and future body indexes, we conducted mediation analyses via the SPSS Macro PROCESS 2.16 (Model 4). In accordance with previous studies [41], Path a denotes the association between the independent variable and the mediator variable; Path b refers to the relationship between the mediator variable and the dependent variable subsequent to adjustment for the independent variable; Path c represents the link between the independent variable and the dependent variable; and Path c′ signifies the link between the independent variable and the dependent variable following adjustment for the mediator variable. The presence of a mediating effect is indicated by the significance of the indirect effect (a × b or c − c′). This study examined the hypothesis that RE mediates the association between brain connectivity and future body indexes (i.e., rsFCs → RE → body indexes). The significance of the mediating effect was evaluated utilizing a bootstrapping method with 5000 iterations.

The raw scores were then converted into z-scores. All analyses were performed utilizing the SPSS 22, with the Greenhouse–Geisser method employed to adjust for *p* value sphericity.

## 3. Results

### 3.1. Behavioral Results

Table 1 shows the descriptive statistics for gender, age, body indexes, degree of hunger and food desire, RE, and NA. The mean values for BMI [*t* (178) = 20.69, *p* < 0.001, *d* = 3.10], RFM [*t* (178) = 9.10, *p* < 0.001, *d* = 1.36], WHtR [*t* (178) = 11.63, *p* < 0.001, *d* = 1.74], and RE [*t* (178) = 5.80, *p* < 0.001, *d* = 0.87] were elevated in overweight group compared to normal-weight group. No group difference was observed in the remaining variables (*p*s > 0.1).

### 3.2. Neural Correlates of Food-Related Intentional Inhibition

To demonstrate the correlation between brain activity and FII, we correlated FII with the ReHo of each voxel across the whole brain. FII was positively associated with ReHo in the left cerebellum (*t* = 3.86, *p* < 0.001), right inferior temporal gyrus (ITG) (*t* = 4.60, *p* < 0.001), right orbitofrontal cortex (OFC) (*t* = 4.56, *p* < 0.001), left inferior frontal gyrus (IFG) (*t* = 5.00, *p* < 0.001), and right cingulate gyrus (*t* = 4.40, *p* < 0.001) (see Figure 1, Table 2).

**Figure 2:**
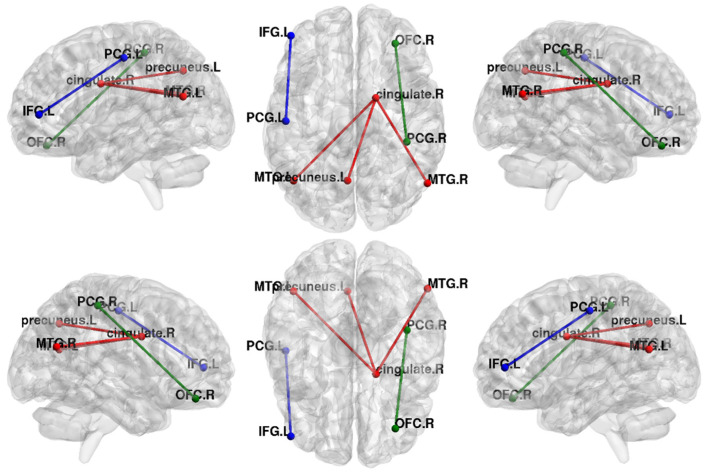
Group differences in rsFCs from the seeds associated with FII. Results showed lower rsFC strength between the IFG and left PCG, and between the left OFC and right PCG in overweight participants versus normal-weight individuals. Results also demonstrated increased rsFC strength between the right cingulate gyrus and bilateral MTG, and between that and the left precuneus in overweight participants versus normal-weight participants. IFG, inferior frontal gyrus; L, left; MFC, medial frontal cortex; MTG, middle temporal gyrus; OFC, orbitofrontal cortex; PCG, postcentral gyrus; R, right. The significance threshold for the identification of regions was set at *p* < 0.05 at the cluster level, combined with *p* < 0.001 at the voxel level, and was two-tailed.

Table 2 also shows the rsFC nodes that were found to be significantly differential between overweight and normal-weight individuals for the cingulate gyrus, OFC, and IFG. Compared to individuals of normal weight, overweight individuals demonstrated decreased connectivity between the left IFG and the left postcentral gyrus (PCG) (*t* = −3.76, *p* < 0.001), and between the left OFC and right PCG (*t* = −4.07, *p* < 0.001). Conversely, overweight individuals exhibited increased connectivity between the right cingulate gyrus and bilateral middle temporal gyrus (MTG) (MTG.R: *t* = 4.19, *p* < 0.001; MTG.L: *t* = 4.56, *p* < 0.001) and between that and the left precuneus (*t* = 4.73, *p* < 0.001) when compared with normal-weight individuals (see Figure 2, Table 2). Using the cerebellum and ITG as seeds, no differences in rsFC were observed between groups. These findings indicated that (1) a close relationship exists between FII and areas related to inhibitory control and maladaptive eating; (2) overweight participants showed decreased connectivity between FII-seeds related to inhibitory control (IFG, OFC) and PCG, as well as increased connectivity between FII-seeds related to maladaptive eating (cingulate) and MTG/precuneus.

### 3.3. Mediation Models

Table 3 provides descriptions highlighting the participants’ body indexes and their changes at the two time points (T1, T2). The associations between the rsFCs and body indexes were investigated through the implementation of linear regression analyses, with adjustments made for gender and age. The results demonstrated that the rsFC between the OFC and right PCG was negatively associated with BMI (T2) (*β* = −0.23, *p* = 0.002) and WHtR (T2) (*β* = −0.30, *p* = 0.003); the rsFCs between the cingulate gyrus and bilateral MTG, and between that and the left precuneus were positively associated with BMI (T2) (cingualte–MTG.R: *β* = 0.20, *p* = 0.007; cingualte–MTG.L: *β* = 0.27, *p* < 0.001; cingualte–precuneus: *β* = 0.21, *p* = 0.004), WHtR (T2) (cingualte–MTG.R: *β* = 0.25, *p* = 0.001; cingualte–MTG.L: *β* = 0.30, *p* < 0.001; cingualte–precuneus: *β* = 0.28, *p* < 0.001), and RFM (T2) (cingualte–MTG.R: *β* = 0.16, *p* = 0.035; cingualte–MTG.L: *β* = 0.22, *p* = 0.004; cingualte–precuneus: *β* = 0.18, *p* = 0.019). In addition, cingulate–precuneus connectivity was negatively associated with ΔRFM (*β* = −0.15, *p* = 0.042) (see Supplementary Table S1).

The results confirmed our third hypothesis, that RE mediated the association between connectivity and future body indexes. Regarding predictions of future body indexes, our results indicate that the negative associations of OFC–PCG connectivity with future body indexes were mediated by RE; the positive associations of connectivity between the cingulate gyrus and MTG/precuneus with future body indexes were mediated by RE. More notably, in predictions of changes in body indexes, our findings indicate that the negative association of cingulate–precuneus connectivity with future change in RFM was totally mediated by RE.

**Figure 3:**
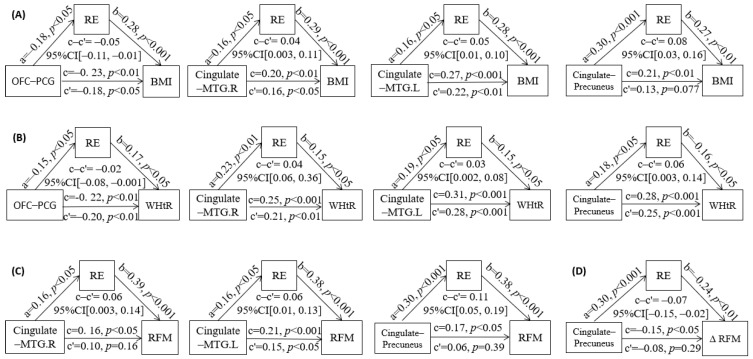
The diagrams demonstrate that, when controlling for FD, gender, and age, RE mediates the relationship between rsFCs (OFC–PCG, cingulate–MTG.R, cingulate–MTG.L, cingulate–precuneus) and (**A**) BMI; (**B**) WHtR; (**C**) between rsFCs (cingulate–MTG.R, cingulate–MTG.L, cingulate–precuneus) and RFM; and (**D**) between cingulate–precuneus connectivity and ΔRFM. BMI, body mass index at T2; RFM, fat mass index at T2; L, left; MTG, middle temporal gyrus; OFC, orbitofrontal cortex; PCG, postcentral gyrus; R, right; RE, restrained eating; WHtR, waist/height ratio at T2; ΔRFM, difference in RFM between T2 and T1 subtraction.

In particular, following the implementation of age and gender as control variables, the 5000 bootstrap simulations indicated that RE exhibited a significant mediating effect on the relationship between rsFC and BMI(T2) (OFC–PCG: c-c′ = −0.05, 95% CI = [−0. 11, −0.01]; cingulate–MTG.R: c-c′ = 0.04, 95% CI = [0.003, 0.11]; cingulate–MTG.L: c-c′ = 0.05, 95% CI = [0.01, 0.10]; cingulate–precuneus: c-c′ = 0.08, 95% CI = [0.03, 0.16]; see Figure 3A); between rsFCs and WHtR (T2) (OFC–PCG: c-c′ = −0.02, 95% CI = [−0.08, −0.001; cingulate–MTG.R: c-c′ = 0.04, 95% CI = [0.06, 0.36]; cingulate–MTG.L: c-c′ = 0.03, 95% CI = [0.002, 0.08]; cingulate–precuneus: c-c′ = 0.03, 95% CI = [0.002, 0.08]; see Figure 3B); between rsFCs and RFM(T2) (cingulate–MTG.R: c-c′ = 0.06, 95% CI = [0.003, 0. 14]; cingulate–MTG.L: c-c′ = 0.06, 95% CI = [0.01, 0.13]; cingulate–precuneus: c-c′ = 0. 11, 95% CI = [0.05, 0.19]; see Figure 3C); and between cingulate–precuneus and ΔRFM (c-c′ = −0.07, 95% CI = [−0.15, −0.02]; see Figure 3D).

## 4. Discussion

This study represented the inaugural endeavor to investigate the neurobiological correlates of food-related intentional inhibition (FII) and its association with future body indexes in two samples of overweight and normal-weight young adults, employing ReHo and brain connectivity as assessed by rs-fMRI. Firstly, whole-brain correlation analyses demonstrated that elevated levels of FII were positively related to ReHo in the left cerebellum, right ITG, right OFC, left IFG, and right cingulate gyrus, which provides new insights into the study of food-specific intentional inhibition. Secondly, we used seed-based rsFC to investigate the different neurobiological representations between overweight and normal-weight participants. The results showed significantly increased rsFC strength between regions associated with FII (IFG, OFC) and bilateral PCG in overweight participants in comparison to normal-weight participants. Concurrently, the findings demonstrated a decrease in rsFC strength between the cingulate gyrus and areas associated with maladaptive eating (MTG, precuneus). Participants also showed that these connectivities predicted body indexes (BMI, RFM, WHtR, ΔRFM) after one year. Furthermore, there may exist models in the relationships between brain connectivity, restrained eating (RE), and body indexes. OFC–PCG connectivity and rsFCs between the cingulate gyrus and areas correlated with maladaptive eating (MTG, precuneus) could be associated with future body indexes (BMI, RFM, WHtR, ΔRFM) and mediated by RE. These findings suggest that the presence of overweight is the result of a multifaceted effect of FII-related neurocognitive processes (e.g., self-control, maladaptive eating) involving brain connectivity and eating behavior (e.g., restrained eating).

### 4.1. Neural Correlates of Food-Specific Intentional Inhibition

This study suggests that decreased ReHo in the right OFC and left IFG is associated with poorer FII performance. Based on a recent study [19], the IFG and OFC have been considered to be involved in inhibitory control. In particular, we employed the ReHo, a widely used rs-fMRI index, to further support the result that IFG and OFC might play a meaningful part in the deduction of inhibitory control in overweight individuals. There are a number of reasons that could account for this association. First, researchers have suggested that IFG is negatively linked to task difficulty and might positively predict future weight gain [17] in the neural study of delay discounting and overweight. Furthermore, the literature has found reduced gray matter in the right IFG of overweight individuals [42], which may suggest that IFG could be an indicator of excess weight. Second, the OFC is a core region for impulsivity, which tends to lead to disinhibitory behavior [43]. Research into obesity has revealed an association between elevated BMI (a consequence of impulsive eating) and increased activation of the OFC [16]. The present study revealed that decreased ReHo in the IFG and OFC may develop as a compensatory mechanism for difficulties in inhibiting food cues, induced by a decline in intentional inhibition in overweight participants.

Furthermore, our findings demonstrated a positive association between FII and ReHo in the cerebellum, MTG, and cingulate gyrus. These regions have been linked to maladaptive eating behaviors [23]. There are several reasons to explain these potential associations between FII and regions associated with maladaptive eating. First, these findings further support prior evidence that the cerebellum is associated with disinhibited eating and hunger [19], indicating that the cerebellum may be an indicator of maladaptive eating and weight gain [18]. Second, the cingulate gyrus is related to food-related expectations and impulsive decision-making [20,21] and is positively linked to BMI [17,24]. A recent longitudinal study found a positive link between the gray matter volume of cingulate gyrus and body fat increase over a period of one to two years [44]. Third, the MTG is considered a critical brain region involved in restrained eating and the executive functions of food intake [22,45], with studies suggesting a potential association with the prevalence of overweight [46]. For instance, research has found that, compared with normal-weight individuals, obese participants exhibit increased neural activation in the MTG when presented with food cues [45]. Evidence from longitudinal studies has suggested that the response of the MTG to food cues can predict future variability in body weight [44], and that the MTG may interact with leptin to control eating behavior during interventions [47]. Thus, functional alterations in the MTG among obese young adults could be critical for identifying imaging-based biomarkers for obesity prevention and treatment [48]. The results suggest that the activation of regions associated with maladaptive eating may lead individuals to experience heightened hunger sensations, as well as increased food cravings and urges. This phenomenon may pose challenges when attempting to intentionally inhibit responses to food cues. Thus, our findings provide more direct evidence supporting the opinion that regions associated with maladaptive eating play a significant role in FII.

### 4.2. Altered Brain Connectivity of FII-Related Region in Overweight Individuals

The present study revealed the altered connectivity between areas correlated with intentional inhibition and maladaptive eating in overweight individuals, which was found in Liu et al. (2022) [18], using food-specific stimuli. Compared to normal-weight individuals, overweight participants showed lower connectivity strength between the seed IFG and the left PCG, and between the seed OFC and right PCG. There are several reasons to explain the connectivity between FII-related regions and the PCG. First, the PCG has been implicated in hunger and disinhibited eating [45]. Previous studies have revealed that an elevated BMI is linked to the PCG [24], which may trigger specific brain responses that lead to a preference for food stimuli and increase the motivation to consume tasty foods [19]. Second, PCG is associated with self-referential processing [49] and depression [50] and is involved with diverse functions related to food reward, eating behaviors, and obesity. Additionally, according to a recent study [14], overweight participants showed increased connectivity between the PCG and pre-SMA, a key region involved in regulating the level of action readiness in situations requiring intentional inhibition [51]. Thus, the decreased connectivity strength between FII-related regions and the PCG in overweight individuals may reflect an increased desire for food stimuli, as well as reduced reactivity to intentional inhibition, which may drive behavior towards food intake.

For the seed cingulate gyrus, we found that overweight participants had increased rsFC values in the bilateral MTG and left precuneus. According to previous research, the MTG and precuneus have been reported as critical regions for the regulation of the executive function [52,53]. First, as mentioned above, the MTG is associated with motivation levels regarding food intake [46]. Evidence from the FC analysis has revealed altered connectivity strength in the cingulate gyrus and temporal lobe network in obese individuals compared to lean individuals [54]. Second, the precuneus is associated with executive function [53], BMI, and eating behavior [55]. Precuneus activity may reflect increased visual attention resulting from increased task difficulty [56] and is linked to decreased body fat and lower hunger levels [55]. In patients with focal brain lesions involving the precuneus, an association between precuneus lesions and impaired executive function has been observed [57]. Based on the literature on FC analysis, we note the enhanced cingulate–precuneus connectivity that was found in both the present study and previous studies [58], which has been associated with higher BMI and eating disorder. Based on the above findings, it is speculated that increased connectivity between the cingulate gyrus and the MTG/precuneus may emerge as a compensation in overweight participants in response to a reduction in or an absence of control over food stimuli. This mechanism may, to some extent, help overweight individuals suppress their motivation to eat.

### 4.3. Prediction of Connectivity on Future Body Indexes: Mediation of RE

The present study showed that RE mediated the effect of rsFCs on body indexes after 1 year. Previous studies have shown that RE positively predicts BMI [59,60,61], while it is negatively related to changes in BMI [62]. These associations were supported in the present study and demonstrated for more body indexes (WHtR; RFM; change in RFM). Thus, RE may be an important dietary factor influencing body weight and its variation.

Findings from fMRI research suggest that activation in the OFC is inversely correlated with activation in the PCG, and increased OFC–PCG connectivity in bulimia nervosa patients has been observed [63]. Moreover, the activation of cingulate gyrus is positively correlated to the activation of the MTG [47] and that of the precuneus [57,58]. Data from cross-sectional and longitudinal studies further support the positive association between cingulate–MTG/precuneus connectivity and BMI [54], as well as future body fat gain [44]. As aforementioned, all of the above regions are related to food-specific inhibitory control or maladaptive eating; RE and body weight are related to food-specific inhibitory control and maladaptive eating [31,32,33]. Therefore, RE may mediate the relationship between these connectivities and future body indexes.

Notably, the present study illustrated that the effects of cingulate–precuneus connectivity on body indexes (BMI/RFM/ΔRFM) were totally mediated by RE. Our results indicate that the cingulate–precuneus connectivity is more closely linked to RE when predicting body indexes. Specifically, RE was positively correlated with BMI, RFM, and WHtR at the one-year follow-up, whilst showing a negative correlation with changes in RFM. These results align with existing evidence that RE positively predicts future body weight and negatively predicts body weight change [59,62]. This contradictory finding may be related to the counter-regulatory effect of RE. When considered collectively, this effect leads individuals to eat more due to concerns about their weight [62], which explains the positive correlation between RE and future body indexes. In this pathway, RE reflects dysregulatory control. However, at an individual level, weight control goals and successful dieting behaviors over a specific period may contribute to weight loss [64] and are therefore negatively correlated with changes in RFM. In this pathway, RE reflects adaptive control. Thus, RE may play a significant role in predicting brain connectivity related to future body indexes.

### 4.4. Food-Specific Intentional Inhibition Model for Overweight

**Figure 4:**
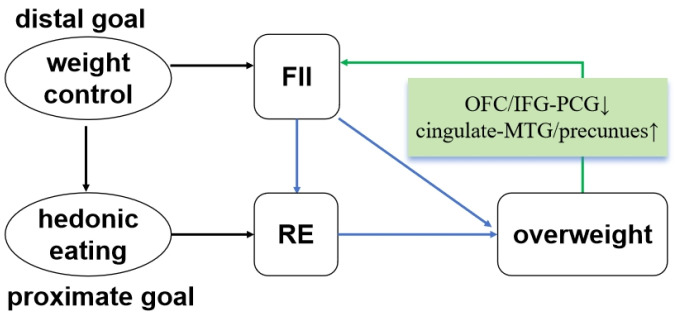
Food-specific intentional inhibition model for overweight. The green lines reflect altered connectivity associated with FII in overweight individuals. The blue lines reflect the mediating pathways through which connectivity predicts future body indexes following RE. The black lines represent elements of the original model not addressed by the evidence from the present study. FII, food-specific intentional inhibition; IFG, inferior frontal gyrus; MTG, middle temporal gyrus; OFC, orbitofrontal cortex; PCG, postcentral gyrus; RE, restrained eating.

To integrate the findings of this study, on the basis of the theoretical intentional inhibition model [7], we propose a food-specific intentional inhibition model for overweight (Figure 4). A detailed explanation is provided below.

Firstly, the distal goal is to control eating behavior through intentional inhibition, thereby reducing the influence of the proximal goal of hedonic eating. Secondly, altered connectivity between regions associated with FII and maladaptive eating suggest that behaviors linked to the proximate goal (hedonic eating) occur frequently in overweight individuals. Consequently, inhibitory control is weakened, necessitating greater compensatory activation in FII-related regions to exert an inhibitory effect. Furthermore, connectivity between brain regions associated with FII and maladaptive eating could predict the long-term effects and dynamic changes associated with being overweight.

Taken together, our model posits that altered connectivity between brain regions involved in FII and those associated with maladaptive eating enables the prediction of changes in overweight status by influencing RE. As overweight/obese individuals typically face a struggle between hedonic eating (proximate goal) and the weight control (distal goal), this model helps to explain the neural basis underlying the difficulty overweight individuals face in achieving FII, as well as the role that RE plays in this process.

### 4.5. Limitations

It is imperative to acknowledge the limitations of this study, which should be considered in future research endeavors. Initially, this study used only ReHo and rsFC as measures of brain function. Therefore, future researchers may consider incorporating alternative imaging metrics to examine the neural basis of food-related intentional inhibition. Secondly, the present design cannot detect causal relationships between variables. Thus, longitudinal designs are required to establish the causal relationships between FII, RE, and overweight in future research. Third, some factors that may affect fMRI signals and food-related inhibitory control, such as habitual diet, sleep, and the menstrual cycle, were not available for our sample. Finally, since the participants in this study were all overweight undergraduates, this may have resulted in a narrow age range, thereby limiting the extent to which the findings can be generalized. Therefore, future research should include a more diverse range of participants, such as obese individuals and adolescents.

## 5. Conclusions

In sum, this study demonstrated: (1) the neural correlates of FII; (2) group differences in functional neural evidence for FII; and (3) that altered connectivity patterns associated with FII predict body indexes 1 year later, and RE plays an important mediating role. Our results suggest a positive association between RE and future body indexes, while indicating a negative correlation between RE and the change in RFM. Based on these findings, we propose a relational model between brain connectivity, RE, and body indexes. Specifically, brain connectivity may influence the future body indexes via RE. Our findings contribute to strengthening the neurobiological foundations of food-related intentional inhibition and the neural mechanisms underlying the relationship between intentional inhibition and excess weight. From the perspective of intentional inhibition, these findings also provide a theoretical basis and neural targets for weight management and interventions for overweight individuals.

## Figures and Tables

**Table 1:** Demographic description of overweight and normal-weight participants.

Variables	Sample 1 (*N* = 100)		Sample 2 (*N* = 180)	
	OW (*n* = 55) *M* ± *SD*	NW (*n* = 45) *M* ± *SD*	OW (*n* = 87) *M* ± *SD*	NW (*n* = 93) *M* ± *SD*
Gender (% male)	27 (49.1%)	24 (53.3%)	37 (42.5%)	40 (43%)
Age (years)	19.26 ± 0.91	19.56 ± 0.69	19. 14 ± 1.12	18.95 ± 0.68
BMI (kg/m^2^)	27.24 ± 1.45	20.34 ± 1.16	26.00 ± 2.10	20.65 ± 1.30
RFM	32.00 ± 6.84	20.60 ± 8.24	31.46 ± 6.44	21.65 ± 7.90
WHtR	0.51 ± 0.05	0.43 ± 0.03	0.50 ± 0.04	0.44 ± 0.03
NA	1.83 ± 0.65	1.9 ± 0.62	2.29 ± 0.36	2.24 ± 0.40
RE	--	--	15.92 ± 4.96	11.37 ± 5.51
Hunger degree	26.07 ± 24.46	27.27 ± 21.75	--	--
Desire to eat	21.88 ± 22.39	28.05 ± 20.80	--	--

Note: *N*, number; OW, overweight group; NW, normal-weight group; *SD*, standard deviation; BMI, body mass index; RFM, relative fat mass; WHtR, waist/height ratio; NA, negative affect; RE, restrained eating.

**Table 2:** Brain regions where ReHo was correlated with FII and rsFC were different between overweight and normal-weight groups.

Region	Side	BA	Peak MNI Coordinates			Peak *t* Value	Cluster Size (mm^3^)
			X	Y	Z		
ReHo correlated with FII							
Cerebellum	L	^#^	−42	−72	−33	3.86	33
ITG	R	20	69	−27	−21	4.60	29
OFC	R	11	33	42	−18	4.56	30
IFG	L	46	−48	48	6	5.00	51
Cingulate	R	^#^	18	0	30	4.40	27
rsFC different between groups							
IFG_(seed region)_–PCG	L	4	−52	−18	50	−3.76	183
OFC_(seed region)_–PCG	R	4	42	−34	54	−4.07	175
Cingulate_(seed region)_–MTG	R	39	58	−66	22	4.19	357
Cingulate_(seed region)_–MTG	L	39	−46	−64	20	4.56	499
Cingulate_(seed region)_–precuneus	L	7	−4	−64	40	4.73	490

Note: BA, Brodmann’s area; FII, food-related intentional inhibition; ITG, inferior temporal gyrus; IFG, inferior frontal gyrus; L, left; MNI, Montreal Neurological Institute; MTG, middle temporal gyrus; OFC, orbitofrontal cortex; PCG, postcentral gyrus; R, right; ReHo, regional homogeneity; rsFC, resting-state functional connectivity. The significance threshold for the identification of regions was set at *p* < 0.05 at the cluster level, combined with *p* < 0.001 at the voxel level, and was two-tailed. ^#^ The absence of a certain area of BA.

**Table 3:** Body indexes of overweight and normal-weight groups at two time points (*N* = 180).

Body Indexes	T1 *M* ± *SD*	T2 *M* ± *SD*	T2–T1 (Δ)	Change Rate (%)
BMI	23.23 ± 3.19	23.78 ± 3.55	0.55	2.37
RFM	26.39 ± 8.73	28.32 ± 8.16	1.93	7.31
WHtR (%)	46.58 ± 4.79	47.77 ± 5.54	1.19	2.55

Note: *N*, number; *SD*, standard deviation; BMI, body mass index; RFM, relative fat mass; WHtR, waist/height ratio; Δ, difference in BMI/RFM/WHtR between T2 and T1.

## Data Availability

The data supporting the conclusions of this article will be made available by the corresponding author on request.

## Supplementary Materials

The following supporting information can be downloaded at: https://www.mdpi.com/article/10.3390/nu18111670/s1, Table S1: Results of model examining the association between connectivity and body indexes change (*N* = 180).

## References

[1] Hemmingsson E. (2021). The unparalleled rise of obesity in China: A call to action. Int. J. Obes..

[2] Farhat T., Iannotti R.J., Caccavale L.J. (2014). Adolescent Overweight, Obesity and Chronic Disease-Related Health Practices: Mediation by Body Image. Obes. Facts.

[3] Song S., Li Q., Jiang Y., Liu Y., Xu A., Liu X., Chen H. (2022). Do Overweight People Have Worse Cognitive Flexibility? Cues-Triggered Food Craving May Have a Greater Impact. Nutrients.

[4] Groppe K., Elsner B. (2015). The influence of hot and cool executive function on the development of eating styles related to overweight in children. Appetite.

[5] Anderson M.C., Crespo-Garcia M., Subbulakshmi S. (2025). Brain mechanisms underlying the inhibitory control of thought. Nat. Rev. Neurosci..

[6] Lynn M.T., Muhle-Karbe P.S., Brass M. (2014). Controlling the self: The role of the dorsal frontomedian cortex in intentional inhibition. Neuropsychologia.

[7] Filevich E., Kuhn S., Haggard P. (2012). Intentional inhibition in human action: The power of ‘no’. Neurosci. Biobehav. Rev..

[8] Stroebe W., van Koningsbruggen G.M., Papies E.K., Aarts H. (2013). Why Most Dieters Fail but Some Succeed: A Goal Conflict Model of Eating Behavior. Psychol. Rev..

[9] Hanlon C.A., Hartwell K.J., Canterberry M., Li X., Owens M., LeMatty T., Prisciandaro J.J., Borckardt J., Brady K.T., George M.S. (2013). Reduction of cue-induced craving through realtime neurofeedback in nicotine users: The role of region of interest selection and multiple visits. Psychiatry Res. Neuroimaging.

[10] Liu Y., Grasman R.P.P.P., Wiers R.W., Ridderinkhof K.R., van den Wildenberg W.P.M. (2020). Moderate acute alcohol use impairs intentional inhibition rather than stimulus-driven inhibition. Psychol. Res..

[11] Liu X., Liu Y., Song S., Xiang G., Du X., Li Q., Xiao M., Ling Y., Chen H. (2022). “Free won’t” of food in overweight and normal-weight adults: Comparison of neurocognitive correlates of intentional and reactive inhibitions. Neuropsychologia.

[12] Shen Y., Wen Y., Gu T., Liu S. (2023). A study of intentional inhibition of food stimuli among female restricted eaters. Appetite.

[13] Si R., Rowe J.B., Zhang J. (2021). Functional localization and categorization of intentional decisions in humans: A meta-analysis of brain imaging studies. Neuroimage.

[14] Schel M.A., Kühn S., Brass M., Haggard P., Ridderinkhof K.R., Crone E.A. (2014). Neural correlates of intentional and stimulus-driven inhibition: A comparison. Front. Hum. Neurosci..

[15] Chen S.Y., Jackson T., Dong D.B., Zhang X.M., Chen H. (2019). Exploring effects of single-session anodal tDCS over the inferior frontal gyrus on responses to food cues and food cravings among highly disinhibited restrained eaters: A preliminary study. Neurosci. Lett..

[16] Coveleskie K., Gupta A., Labus J.S., Mayer D., Ashe-McNalley C., Stains J., Kilpatrick L.A., Mayer E.A. (2014). Sa1364 Altered Resting State and Functional Connectivity Between the Nucleus Accumbens and Reward-Based Regions in Overweight and Obese Women. Gastroenterology.

[17] Kishinevsky F.I., Cox J.E., Murdaugh D.L., Stoeckel L.E., Cook E.W., Weller R.E. (2012). fMRI reactivity on a delay discounting task predicts weight gain in obese women. Appetite.

[18] Liu X., Chen X., Li Q., Xiang G., Li W., Xiao M., Du X., Song S., Liu Y., Chen H. (2022). Altered resting-state functional connectivity ofmedial frontal cortex in overweight individuals: Link to food-specific intentional inhibition and weight gain. Behav. Brain Res..

[19] Syan S.K., McIntyre-Wood C., Minuzzi L., Hall G., McCabe R.E., MacKillop J. (2021). Dysregulated resting state functional connectivity and obesity: A systematic review. Neurosci. Biobehav. Rev..

[20] Simon J.J., Skunde M., Walther S., Bendszus M., Herzog W., Friederich H.C. (2016). Neural signature of food reward processing in bulimic-type eating disorders. Soc. Cogn. Affect. Neurosci..

[21] Peters J., Buechel C. (2009). Overlapping and Distinct Neural Systems Code for Subjective Value during Intertemporal and Risky Decision Making. J. Neurosci..

[22] Zhang P., Wu G., Yu F., Liu Y., Li M., Wang Z., Ding H., Li X., Wang H., Jin M. (2020). Abnormal Regional Neural Activity and Reorganized Neural Network in Obesity: Evidence from Resting-State fMRI. Obesity.

[23] Hampton-Anderson J.N., Craighead L.W. (2021). Psychosociocultural Contributors to Maladaptive Eating Behaviors in African American Youth: Recommendations and Future Directions. Am. J. Lifestyle Med..

[24] Chao S.H., Liao Y.T., Chen V.C.H., Li C.J., McIntyre R.S., Lee Y., Weng J.C. (2018). Correlation between brain circuit segregation and obesity. Behav. Brain Res..

[25] Fox M.D., Raichle M.E. (2007). Spontaneous fluctuations in brain activity observed with functional magnetic resonance imaging. Nat. Rev. Neurosci..

[26] Zuo X.N., Xu T., Jiang L., Yang Z., Cao X.Y., He Y., Zang Y.F., Castellanos F.X., Milham M.P. (2013). Toward reliable characterization of functional homogeneity in the human brain: Preprocessing, scan duration, imaging resolution and computational space. Neuroimage.

[27] Zang Y., Jiang T., Lu Y., He Y., Tian L. (2004). Regional homogeneity approach to fMRI data analysis. Neuroimage.

[28] Govindarajulu Z. (1992). Rank correlation methods. Technometrics.

[29] Wu T., Long X., Zang Y., Wang L., Hallett M., Li K., Chan P. (2009). Regional homogeneity changes in patients with Parkinson’s disease. Hum. Brain Mapp..

[30] Agarwal S., Sair H.I., Pillai J.J. (2017). The Resting-State Functional Magnetic ResonanceImaging Regional Homogeneity Metrics-Kendall’s Coefficient of Concordance-Regional Homogeneity and Coherence-Regional Homogeneity-Are Valid Indicators of Tumor-Related Neurovascular Uncoupling. Brain Connect..

[31] Mayer M.A., Catalani F., Fraire J., Deltetto N., Martín L., Beneitez A., Fischman D., Orden A.B. (2022). Inhibitory control and obesity in adolescents: A prospective cohort study. Appetite.

[32] Loeber S., Rustemeier M., Paslakis G., Pietrowsky R., Mueller A., Herpertz S. (2018). Mood and restrained eating moderate food-associated response inhibition in obese individuals with binge eating disorder. Psychiatry Res..

[33] Price M., Lee M., Higgs S. (2016). Food-specific response inhibition, dietary restraint and snack intake in lean and overweight/obese adults: A moderated-mediation model. Int. J. Obes..

[34] Suthahar N., Bergman R.N., de Boer R.A. (2025). Replacing body mass index with relative fat mass to accurately estimate adiposity. Nat. Rev. Endocrinol..

[35] Watson D., Clark L.A., Tellegen A. (1988). Development and validation of brief measures of positive and negative affect—The PANAS scales. J. Personal. Soc. Psychol..

[36] Kong F.C., Zhang Y., Chen H. (2013). The construct validity of the Restraint Scale among mainland Chinese women. Eat. Behav..

[37] Caparelli E.C., Ross T.J., Gu H., Yang Y. (2019). Factors Affecting Detection Power of Blood Oxygen-Level Dependent Signal in Resting-State Functional Magnetic Resonance Imaging Using High-Resolution Echo-Planar Imaging. Brain Connect..

[38] Krishnamurthy V., Gopinath K., Brown G.S., Hampstead B.M. (2015). Resting-state fMRI reveals enhanced functional connectivity in spatial navigation networks after transcranial direct current stimulation. Neurosci. Lett..

[39] Pellegrino G., Schuler A.L., Arcara G., Di Pino G., Piccione F., Kobayashi E. (2022). Resting state network connectivity is attenuated by fMRI acoustic noise. Neuroimage.

[40] Andretta J.R., McKay M.T. (2020). Self-efficacy and well-being in adolescents: A comparative study using variable and person-centered analyses. Child. Youth Serv. Rev..

[41] Xiang G., Li Q., Xiao M., He L., Chen X., Du X., Liu X., Song S., Wu Y., Chen H. (2021). Goal setting and attaining: Neural correlates of positive coping style and hope. Psychophysiology.

[42] Herrmann M.J., Tesar A.K., Beier J., Berg M., Warrings B. (2019). Grey matter alterations in obesity: A meta-analysis of whole-brain studies. Obes. Rev..

[43] Mavrogiorgou P., Enzi B., Klimm A.K., Köhler E., Roser P., Norra C., Juckel G. (2017). Serotonergic modulation of orbitofrontal activity and its relevance for decision making and impulsivity. Hum. Brain Mapp..

[44] Winter S.R., Yokum S., Stice E., Osipowicz K., Lowe M.R. (2017). Elevated reward response to receipt of palatable food predicts future weight variability in healthy-weight adolescents. Am. J. Clin. Nutr..

[45] Zhao J., Li M., Zhang Y., Song H., von Deneen K.M., Shi Y., Liu Y., He D. (2017). Intrinsic brain subsystem associated with dietary restraint, disinhibition and hunger: An fMRI study. Brain Imaging Behav..

[46] Yokum S., Stice E. (2019). Weight gain is associated with changes in neural response to palatable food tastes varying in sugar and fat and palatable food images: A repeated-measures fMRI study. Am. J. Clin. Nutr..

[47] Li Z., Wu X., Gao H., Xiang T., Zhou J., Zou Z., Tong L., Yan B., Zhang C., Wang L. (2023). Intermittent energy restriction changes the regional homogeneity of the obese human brain. Front. Neurosci..

[48] Zhao J., Long Z., Li Y., Qin Y., Liu Y. (2022). Alteration of regional heterogeneity and functional connectivity for obese undergraduates: Evidence from resting-state fMRI. Brain Imaging Behav..

[49] Li G., Ji G., Hu Y., Xu M., Jin Q., Liu L., von Deneen K.M., Zhao J., Chen A., Cui G. (2018). Bariatric surgery in obese patients reduced resting connectivity of brain regions involved with self-referential processing. Hum. Brain Mapp..

[50] Liu C.H., Li F., Li S.F., Wang Y.J., Tie C.L., Wu H.Y., Zhou Z., Zhang D., Dong J., Yang Z. (2012). Abnormal baseline brain activity in bipolar depression: A resting state functional magnetic resonance imaging study. Psychiatry Res. Neuroimaging.

[51] Passingham R.E., Bengtsson S.L., Lau H.C. (2010). Medial frontal cortex: From self-generated action to reflection on one’s own performance. Trends Cogn. Sci..

[52] Sui C., Wen H., Han J., Chen T., Gao Y., Wang Y., Yang L., Guo L. (2023). Decreased gray matter volume in the right middle temporal gyrus associated with cognitive dysfunction in preeclampsia superimposed on chronic hypertension. Front. Neurosci..

[53] Dadario N.B., Sughrue M.E. (2023). The functional role of the precuneus. Brain.

[54] Kullmann S., Heni M., Veit R., Ketterer C., Schick F., Haering H.U., Fritsche A., Preissl H. (2012). The obese brain: Association of body mass index and insulin sensitivity with resting state network functional connectivity. Hum. Brain Mapp..

[55] McFadden K.L., Cornier M.A., Melanson E.L., Bechtell J.L., Tregellas J.R. (2013). Effects of exercise on resting-state default mode and salience network activity in overweight/obese adults. Neuroreport.

[56] Barber A.D., Carter C.S. (2005). Cognitive control involved in overcoming prepotent response tendencies and switching between tasks. Cereb. Cortex.

[57] Yeager B.E., Bruss J., Duffau H., Herbet G., Hwang K., Tranel D., Boes A.D. (2022). Central precuneus lesions are associated with impaired executive function. Brain Struct. Funct..

[58] Lee S., Kim K.R., Ku J., Lee J.H., Namkoong K., Jung Y.C. (2013). Resting-state synchrony between anterior cingulate cortex and precuneus relates to body shape concern in anorexia nervosa and bulimia nervosa. Psychiatry Res..

[59] Snoek H.M., Engels R.C.M.E., van Strien T., Otten R. (2013). Emotional, external and restrained eating behaviour and BMI trajectories in adolescence. Appetite.

[60] Snoek H.M., van Strien T., Janssens J.M.A.M., Engels R.C.M.E. (2008). Restrained Eating and BMI: A Longitudinal Study Among Adolescents. Health Psychol..

[61] Lowe M.R., Doshi S.D., Katterman S.N., Feig E.H. (2013). Dieting and restrained eating as prospective predictors of weight gain. Front. Psychol..

[62] Schaumberg K., Anderson D.A., Anderson L.M., Reilly E.E., Gorrell S. (2016). Dietary restraint: What’s the harm? A review of the relationship between dietary restraint, weight trajectory and the development of eating pathology. Clin. Obes..

[63] Li L., Yu H., Zhong M., Liu S., Wei W., Meng Y., Li M.-L., Li T., Wang Q. (2022). Gray matter volume alterations in subjects with overweight and obesity: Evidence from a voxel-based meta-analysis. Front. Psychiatry.

[64] Liu Y., Zhang L.L., Jackson T., Wang J.M., Yang R.L., Chen H. (2020). Effects of negative mood state on event-related potentials of restrained eating subgroups during an inhibitory control task. Behav. Brain Res..

