# Supplementary material for: Effects of Neural Correlates of Food-Specific Intentional Inhibition in Predicting Body Fat Loss for Overweight and Normal-Weight Young Adults: The Mediation of Restrained Eating
Source: Nutrients. 2026 May 23;18(11):1670. doi: 10.3390/nu18111670 (PMC13259295; doi:10.3390/nu18111670)
Supplement: Supplementary file 1 [file nutrients-18-01670-s001.zip › nutrients-4295839-supplementary.pdf]

Supplementary Table S1. Results of model examining the association between connectivity and body indexes change (*N*=180)

Body indexes	Independent variables	*R*	R^2	ΔR^2	*F*	*β*	*t(p)*
	Step 1						
BMI(T2)	sex, age	0.01	<0.001	-0.005	0.02		
	Step 2						
	OFC-PCG	0.23	0.05	0.04	4.89	-0.23	-3.12(0.002)
	cingulate-MTG.R	0.20	0.04	0.03	3.74	0.20	2.73(0.007)
	cingulate-MTG.L	0.27	0.07	0.06	6.87	0.27	3.70(<0.001)
	cingulate-precuneus	0.21	0.05	0.04	4.20	0.21	2.90(0.004)
	Step 1						
WHtR(T2)	sex, age	0.05	0.003	-0.003	0.47		
	Step 2						
	OFC-PCG	0.23	0.05	0.04	4.75	-0.22	-3.00(0.003)
	cingulate-MTG.R	0.25	0.06	0.05	5.94	0.25	3.37(0.001)
	cingulate-MTG.L	0.31	0.10	0.09	9.31	0.30	4.25(<0.001)
	cingulate-precuneus	0.28	0.08	0.07	7.55	0.28	3.82(<0.001)
	Step 1						
FMI(T2)	sex, age	0.09	0.01	0.002	1.41		
	Step 2						
	cingulate-MTG.R	0.18	0.03	0.02	2.97	0.16	2.12(0.035)
	cingulate-MTG.L	0.23	0.05	0.04	5.07	0.22	2.94(0.004)
	cingulate-precuneus	0.20	0.04	0.03	3.54	0.18	2.38(0.019)
	Step 1						
ΔFMI	sex, age	0.08	0.01	<0.001	1.05		
	Step 2						
	cingulate-precuneus	0.17	0.03	0.02	2.63	-0.15	-2.05(0.042)

Note: BMI(T2), body mass index at T2; WHtR(T2), Waist/height ratio at T2; FMI(T2), fat mass index at T2; ΔFMI, difference in FMI between T2 and T1 subtraction; OFC, orbitofrontal cortex; PCG, postcentral gyrus; MTG, middle temporal gyrus; R, right; L, left.
